# Supplementary material for: Correlation between relative afferent pupillary defect and visual field defects on Humphrey automated perimetry: A cross-sectional clinical trial
Source: PLoS One. 2022 May 26;17(5):e0267469. doi: 10.1371/journal.pone.0267469 (PMC9135214; doi:10.1371/journal.pone.0267469)
Supplement: Attachment — Submitted filename: Response to Reviewers.docx [file pone.0267469.s003.docx]

PONE-D-21-21722

**Response to Reviewers**

**Correlation between relative afferent pupillary defect and visual field defects on Humphrey automated perimetry: A cross-sectional clinical trial**

Juthamat Witthayaweerasak, MD; Pemika Lertjittham, MD; Nipat Aui-aree, MD

We sincerely appreciate the efforts of the editor and reviewers in the thorough review of our manuscript. We believe that the suggested changes have greatly improved our article, and we hope it is now suitable for publication in *PLOS ONE*. We have addressed all the journal requirements and the valuable comments of the editor and reviewers below. Our revisions in accordance with the suggestions made in the comments are shown with track changes in the revised manuscript.

**Journal Requirements:**

When submitting your revision, we need you to address these additional requirements.

1. Please ensure that your manuscript meets PLOS ONE's style requirements, including those for file naming. The PLOS ONE style templates can be found at https://journals.plos.org/plosone/s/file?id=wjVg/PLOSOne_formatting_sample_main_body.pdf and <https://journals.plos.org/plosone/s/file?id=ba62/PLOSOne_formatting_sample_title_authors_affiliations.pdf>

**Response**: We have ensured that the manuscript has been formatted per guidelines.

2. Thank you for stating the following financial disclosure: "JW. was supported by a grant from Faculty of Medicine, Prince of Songkla University, Songkhla, Thailand. The funders had no role in study design, data collection and analysis, decision to publish, or preparation of the manuscript."

We note that one or more of the authors is affiliated with the funding organization, indicating the funder may have had some role in the design, data collection, analysis or preparation of your manuscript for publication; in other words, the funder played an indirect role through the participation of the co-authors. If the funding organization did not play a role in the study design, data collection and analysis, decision to publish, or preparation of the manuscript and only provided financial support in the form of authors' salaries and/or research materials, please do the following:

a. Review your statements relating to the author contributions, and ensure you have specifically and accurately indicated the role(s) that these authors had in your study. These amendments should be made in the online form.

b. Confirm in your cover letter that you agree with the following statement, and we will change the online submission form on your behalf: “The funder provided support in the form of salaries for authors [insert relevant initials], but did not have any additional role in the study design, data collection and analysis, decision to publish, or preparation of the manuscript. The specific roles of these authors are articulated in the ‘author contributions’ section.

**Response**: We agree with the statements and have included relevant information in the cover letter accordingly.

3. We note that you have stated that you will provide repository information for your data at acceptance. Should your manuscript be accepted for publication, we will hold it until you provide the relevant accession numbers or DOIs necessary to access your data. If you wish to make changes to your Data Availability statement, please describe these changes in your cover letter and we will update your Data Availability statement to reflect the information you provide.

**Response**: Our research data are accessible using the following link: <https://doi.org/10.6084/m9.figshare.19389680.v1>

4. Please include captions for your Supporting Information files at the end of your manuscript, and update any in-text citations to match accordingly. Please see our Supporting Information guidelines for more information: <http://journals.plos.org/plosone/s/supporting-information>.

**Response**: We have included the captions for our supporting information filed at the end of our manuscript (pages 20, line 385-386).

**Editor Comments:**

1. Add the n to table 1: eyes with and without RAPD

**Response:** Thank you for your valuable advice. Your suggestion has been reflectd in Table 2, as shown below. Please note that Table 1 of the old manuscript has been revised to Table 2 in the revised draft. We would also like to clarify that the terms “Eyes with RAPD” and “Eyes without RAPD” have been revised as “RAPD-positive eyes” and “Contralateral eyes,” respectively, in the revised version of the manuscript.

**Table 2.** Visual acuity and visual field parameters between RAPD-positive eyes and contralateral eyes

| **Parameters** | **RAPD-positive eyes (n=52)** | | **Contralateral eyes (n=52)** | | ***P*-value** |
| --- | --- | --- | --- | --- | --- |
|  | Mean ± SD | Mean (95% CI)* | Mean ± SD | Mean (95% CI)* |  |
| Visual acuity (logMAR) | 0.31 ± 0.30 | 0.31 (0.25, 0.38) | 0.12 ± 0.13 | 0.12 (0.06, 0.18) | <0.001 |
| Mean deviation (dB) | −13.10 ± 7.73 | −13.10 (−14.75, −11.46) | −2.95 ± 3.86 | −2.95 (−4.60, −1.31) | <0.001 |
| Pattern standard deviation (dB) | 9.97 ± 4.53 | 9.97 (8.80, 11.14) | 3.90 ± 4.12 | 3.90 (2.73, 5.06) | <0.001 |
| Visual field index (%) | 64.38 ± 24.22 | 64.38 (59.35, 69.42) | 93.90 ± 10.68 | 93.90 (88.86, 98.94) | <0.001 |

2. Mention how many patients had RAPD and their grades by clinical plus criteria, and how many patients had RAPD by neutral density filters, and their mean grading.

**Response:** Thank you for your insightful suggestion. All patients with RAPD were categorized into three groups using the clinical plus scale and six groups using the neutral density filter, and this information has been mentioned in the results section (page 9, lines 165-166, Table 1).

**Comments to the Authors**

1. Is the manuscript technically sound, and do the data support the conclusions?

The manuscript must describe a technically sound piece of scientific research with data that supports the conclusions. Experiments must have been conducted rigorously, with appropriate controls, replication, and sample sizes. The conclusions must be drawn appropriately based on the data presented.

Reviewer #1: Yes

Reviewer #2: Yes

2. Has the statistical analysis been performed appropriately and rigorously?

Reviewer #1: Yes

Reviewer #2: I Don’t Know

3. Have the authors made all data underlying the findings in their manuscript fully available?

The PLOS Data policy requires authors to make all data underlying the findings described in their manuscript fully available without restriction, with rare exception (please refer to the Data Availability Statement in the manuscript PDF file). The data should be provided as part of the manuscript or its supporting information, or deposited to a public repository. For example, in addition to summary statistics, the data points behind means, medians and variance measures should be available. If there are restrictions on publicly sharing data—e.g. participant privacy or use of data from a third party—those must be specified.

Reviewer #1: No

Reviewer #2: Yes

**Response**: Our research data are accessible using the following link: <https://doi.org/10.6084/m9.figshare.19389680.v1>

4. Is the manuscript presented in an intelligible fashion and written in standard English?
   PLOS ONE does not copyedit accepted manuscripts, so the language in submitted articles must be clear, correct, and unambiguous. Any typographical or grammatical errors should be corrected at revision, so please note any specific errors here.

Reviewer #1: Yes

Reviewer #2: Yes

5. Review comments to the Author

**Reviewer #1**

**Comment**

*This is a straightforward and useful trial. However, the authors did not include much information about the enrolled patient demographics. Please provide a table with the subject characteristics (age, sex, diagnosis, monocular vs binocular disease, visual acuity, etc.) and details on how these related to their study-related outcomes of RAPD and HVF metrics.*

**Response:** Thank you for your thoughtful suggestion. Demographic data and baseline characteristics have been added in the results section (page 8, lines 153-154, Table 1) and in the discussion section (page 13, lines 237-245) as follows:

**Results:** Demographic data and baseline characteristics are summarized in Table 1.

**Table 1.** Demographic data and baseline characteristics

| **Factors** | **n (%)** |
| --- | --- |
| Gender  Male  Female | 24 (46.2)  28 (53.8) |
| Clinical diagnosis of affected eye  Compressive optic neuropathy  Anterior ischemic optic neuropathy  Glaucoma  Optic neuritis  Traumatic optic neuropathy  Other causes | 21 (40.4)  10 (19.2)  8 (15.4)  7 (13.5)  2 (3.8)  4 (7.7) |
| RAPD by clinical plus scale  1+  2+  3+ | 10 (19.2)  21 (40.4)  21 (40.4) |
| RAPD by neutral density filter (log unit)  0.3  0.6  0.9  1.2  1.5  1.8 | 2 (3.8)  8 (15.4)  8 (15.4)  8 (15.4)  5 (9.6)  21 (40.4) |

**Discussion**: Clinical identification of RAPD using the swinging light test is sensitive for detecting optic nerve disorders or substantial retinal disease. In our study, we assessed the quantification of RAPD in patients who had various etiologies of optic neuropathy or retinopathy, such as retinal artery occlusion and retinal detachment. Regarding the baseline characteristics, most patients had severe RAPD using the clinical plus scale (grade 2+ to 3+) and neutral density filters (1.8 log unit). The average Humphrey VFI of the RAPD-positive eyes was 64.4%, indicating profound visual field loss, while the mean logMAR visual acuity was 0.3, considered nearly normal vision. Therefore, RAPD may be present in patients with normal visual acuity who had suffered significant visual field defects.

.

***Reviewer #2***

***Comment 1***

*How may you justify the absence of RAPD due to symmetric optic nerve damage?*

**Response:** As mentioned in the methods section, we enrolled patients who had RAPD determined using the swinging light test performed by two independent investigators. In case of discrepancy, the RAPD test was conducted, and the results were reviewed by a second neuro-ophthalmologist (N.A.) to reach a consensus.

***Comment 2***

*The presence of RAPD can correlate with the damage to RNFL in the presence of unilateral optic nerve disease but can not justify the importance of visual field in relation to visual pathway disorders. It will be good to mention that in the paper. Also, visual field plays a fundamental role for diagnosis of visual pathway disorder.*

**Response**: Thank you for your valuable suggestion. As advised, we have mentioned the importance of the visual field in the discussion section (pages 13, lines 225-227 and page 16, lines 298-301) as follows:

“Although evaluation of the visual field using Humphrey automated perimetry plays a fundamental role in diagnosing visual pathway disorders and the swinging light test with neutral density filters can be used to assess the severity of visual function abnormalities, the facilities for these tests are not always available at clinics.”

“Additionally, the presence of RAPD can correlate with damage to the retinal nerve fiber layers in the presence of unilateral optic nerve disease but cannot justify the importance of the visual field in relation to visual pathway disorders”
